# Ronald Ross: Pioneer of Malaria Research and Nobel Laureate

**DOI:** 10.7759/cureus.65993

**Published:** 2024-08-02

**Authors:** Vineeta Pande, Mridu Bahal, Jasleen Dua, Aryan Gupta

**Affiliations:** 1 Pediatrics, Dr. D.Y. Patil Medical College, Hospital and Research Center, Pune, IND; 2 Pediatric Neurology, Dr. D.Y. Patil Medical College, Hospital and Research Center, Pune, IND

**Keywords:** medical laboratory sciences education, parasite infection, mosquito vector, ronald ross, malaria research

## Abstract

Sir Ronald Ross, a British medical doctor and researcher, is renowned for his pioneering work in malaria research. His discovery of the malaria parasite's lifecycle within mosquitoes revolutionized the understanding and control of malaria, transitioning the field from the miasma theory to vector-based strategies. This literature review aims to explore the comprehensive contributions of Ronald Ross to malaria research and their enduring impact on public health.

## Introduction and background

Sir Ronald Ross's seminal discovery in 1897 of the malaria parasite in the stomach tissue of Anopheles mosquitoes provided the first conclusive evidence that mosquitoes are vectors of malaria [1]. Beyond his contributions to tropical medicine, Ross was also a poet and writer, and his work in mathematical modeling of disease transmission laid the groundwork for modern epidemiology [2]. His pioneering efforts have had a profound and lasting impact on global health, particularly in the fight against malaria.

## Review

Methods

Eligibility Criteria

Studies and historical records focusing on Ronald Ross's work on malaria were included. Exclusion criteria involved unrelated medical studies and general historical accounts without a focus on malaria.

Information Sources

Databases searched included PubMed, JSTOR, and historical archives from the Royal Society and the London School of Hygiene and Tropical Medicine.

Search Strategy

Keywords used were "Ronald Ross," "malaria research," "mosquito transmission," and "malaria history." Searches were conducted from January 2023 to June 2023.

Study Selection

Titles and abstracts were screened for relevance, followed by full-text reviews to confirm inclusion.

Data Extraction

Key data points included the nature of Ross's discoveries, the methodologies used, and the impact on malaria control.

Results

Early Life and Education

Ronald Ross was born on May 13, 1857, in Almora, India, where his father served in the British Army. Despite initially showing more interest in literature and mathematics, Ross completed his medical studies and joined the Indian Medical Service in 1881 [3]. His father, Sir Campbell Claye Grant Ross, was a general in the British Indian Army, and his mother, Matilda Charlotte Elderton, came from a well-established family. Growing up in a family with a strong military and colonial background exposed Ross to diverse cultures and environments from a young age. Ross spent his early years in India before being sent to England for his education at the age of eight [4]. He attended several schools, including Ryde School on the Isle of Wight and Springhill House in Southampton. His schooling laid a strong foundation for his later academic pursuits. During this time, Ross developed a keen interest in literature and the arts, writing poems and plays showcasing his creative abilities. Initially inclined toward a career in the arts, Ross's interest in medicine was sparked by a fascination with science and biology. Influenced by his father’s discipline and his mother’s encouragement, he gradually gravitated toward the medical field. A turning point came when he observed the effects of tropical diseases during his time in India, fueling his desire to find solutions to these health challenges. Ross enrolled at St Bartholomew's Hospital Medical College in London in 1875. Despite struggling with the rigid structure of formal medical education, he excelled in practical subjects and showed a particular interest in parasitology. His mentors, including Dr. Samuel Gee, played a crucial role in guiding his medical training and research focus. He graduated in 1879, becoming a member of the Royal College of Surgeons. After completing his education, Ross joined the Indian Medical Service in 1881. His postings in various parts of India exposed him to numerous medical challenges, particularly malaria. His early career involved treating patients, conducting research, and gathering data on the disease, which later proved invaluable in his groundbreaking work. Ross's perseverance and innovative thinking during these formative years laid the groundwork for his future discoveries [5].

Path to Discovery

Ross's interest in malaria was piqued during his service in India, where the disease was rampant. At the time, the cause of malaria was a subject of intense scientific debate. Ross's initial research did not focus on malaria but on various medical issues, including public health and bacteriology. However, his appointment to the Indian Medical Service in 1881 marked the beginning of his lifelong association with malaria research [6]. In India, Ross became increasingly interested in the disease, which was a significant cause of morbidity and mortality among soldiers and civilians alike. His encounter with Sir Patrick Manson, often referred to as the "father of tropical medicine," was a turning point. Manson proposed the hypothesis that malaria was transmitted by mosquitoes, a theory that Ross would later confirm. Manson's guidance and encouragement were crucial in steering Ross toward his groundbreaking research on malaria transmission [6]. His work was characterized by meticulous experimentation and an unwavering commitment to uncovering the truth. Ross's research involved dissecting thousands of mosquitoes to study the development of the malaria parasite within them. In 1897, Ross's perseverance paid off. He discovered the presence of the malaria parasite, Plasmodium, in the stomach of an Anopheles mosquito that had fed on an infected patient [1]. This finding was a monumental breakthrough, confirming that mosquitoes were indeed the vectors of malaria. Ross's work provided a crucial link in understanding the transmission cycle of the disease, a discovery that revolutionized the field of tropical medicine.

Landmark Discoveries

In 1897, while working in Secunderabad, India, Ross made a critical breakthrough. He discovered the presence of malaria parasites in the stomach tissue of Anopheles mosquitoes that had fed on malaria-infected patients. This finding provided the definitive evidence needed to confirm that mosquitoes were vectors for malaria transmission. On August 20, 1897, Ross identified the complete lifecycle of the malaria parasite in mosquitoes, demonstrating that the parasites developed in the mosquito's stomach and migrated to its salivary glands [1]. This discovery provided irrefutable evidence that malaria was transmitted through the bite of infected mosquitoes, thus revolutionizing our understanding of the disease and laying the groundwork for future control strategies. Nobel Prize and Recognition Ross's seminal work earned him the Nobel Prize in Physiology or Medicine in 1902. His discoveries were instrumental in the development of strategies to combat malaria, including mosquito control and public health campaigns to reduce mosquito breeding grounds. Ross's work laid the foundation for future research on malaria and other vector-borne diseases [7].

Mathematical Modeling of Disease Transmission 

Ross's work on the malaria parasite's life cycle involved not only detailed microscopic observations but also an understanding of the broader ecological and epidemiological implications. He was among the first to apply a holistic approach to studying infectious diseases, considering factors such as the behavior and habitat of mosquito vectors [2,8]. This interdisciplinary perspective was ahead of its time, integrating entomology, parasitology, and epidemiology in a manner that set new standards for scientific research. Ronald Ross's work on the life cycle of the malaria parasite and its transmission by mosquitoes provided the empirical foundation for his interest in mathematical modeling. After identifying the mosquito as the vector for malaria, Ross recognized the need for a theoretical framework to understand and predict the spread of the disease within populations. His background in mathematics and his systematic approach to scientific research naturally led him to develop mathematical models to describe malaria transmission. Ross's models were based on the fundamental principles of population dynamics and infectious disease transmission. He sought to quantify the relationship between mosquito population density, human infection rates, and environmental factors influencing both mosquito behavior and human exposure. This approach was innovative for its time, integrating biological, environmental, and epidemiological data into a cohesive mathematical framework. The implications of Ross's findings were profound. By establishing the role of mosquitoes in malaria transmission, Ross's research led to significant changes in public health policies and practices [4]. Efforts to control mosquito populations through insecticides, environmental management, and the use of bed nets have saved countless lives and continue to be central to malaria prevention programs worldwide.

The Basic Structure of the Model

Ross's mathematical model of malaria transmission, often referred to as the "Ross model," was one of the earliest attempts to describe the dynamics of an infectious disease using differential equations. The model's primary components included human population (H): representing the number of susceptible, infected, and recovered individuals in the human population; mosquito population (M): representing the number of mosquitoes capable of transmitting the malaria parasite; and transmission rates: parameters describing the rate at which mosquitoes bite humans and the probability of transmitting the parasite during a bite [9].

Key equations and parameters: The Ross model utilized a set of differential equations to describe the changes in the number of infected humans and mosquitoes over time. Key parameters in the model included biting rate (a): the average number of bites per mosquito per day; transmission probability (b): the probability of a mosquito becoming infected after biting an infected human; recovery rate (r): the rate at which infected humans recover from the infection; and mortality rate (μ): the natural death rate of mosquitoes.

The core equations of the Ross model can be summarized as follows: human infection dynamics: dI_H_/dt=a⋅b⋅M⋅S_H_−r⋅I_H_, where I_H _is the number of infected humans, S_H_ is the number of susceptible humans, and M is the number of mosquitoes. Mosquito infection dynamics: dI_M_/dt=a⋅c⋅I_H_⋅(M−I_M_)−μ⋅I_M_, where I_M_ is the number of infected mosquitoes, and c is the probability of a human becoming infected after being bitten by an infected mosquito [10].

Insights From the Model

Ross's model provided several critical insights into malaria transmission: The model identified threshold conditions for malaria transmission, suggesting that malaria could only persist in a population if the number of mosquitoes and the biting rate exceeded certain critical values. This concept laid the groundwork for the idea of the "basic reproduction number" (R0), a key parameter in modern epidemiology that indicates the average number of secondary infections produced by an infected individual in a fully susceptible population. Ross's model demonstrated that reducing mosquito populations or interrupting the mosquito-human transmission cycle could significantly reduce the incidence of malaria. This insight underscored the importance of vector control measures, such as insecticide spraying and environmental management, in malaria prevention [8].

Advanced developments and extensions: Ross's pioneering work in mathematical modeling inspired subsequent researchers to develop more sophisticated models of disease transmission [9,11-13]. His approach to integrating empirical data with theoretical models set a precedent for future epidemiological studies. Researchers built upon Ross's foundational work, incorporating additional complexities such as varying human immunity, heterogeneous mosquito populations, and spatial dynamics of disease spread [14-16]. The principles and methods introduced by Ross were not limited to malaria. They were adapted and extended to study the transmission dynamics of other vector-borne diseases, such as dengue fever, Zika virus, and filariasis [11,17]. Ross's emphasis on understanding the ecological and behavioral aspects of disease vectors became a cornerstone of vector-borne disease modeling. Modern epidemiological models have become increasingly sophisticated, incorporating advanced statistical methods, computational techniques, and vast datasets. However, the core principles established by Ross remain integral to the field. Contemporary models often use Ross's framework as a starting point, refining and expanding it to address current public health challenges.

Later Career and Contributions

After his groundbreaking work in India, Ross returned to England and continued his research and advocacy in tropical medicine. He held prominent positions, including a professorship at the Liverpool School of Tropical Medicine and later at King's College Hospital in London. Ross also contributed to the mathematical modeling of malaria transmission, further enhancing the understanding of the disease dynamics.

Legacy

Ronald Ross's contributions to science and medicine extend beyond his discoveries about malaria [5]. His interdisciplinary approach, combining field research, laboratory work, and mathematical modeling, set a precedent for future scientific investigations. Ross's legacy is reflected in the ongoing global efforts to eradicate malaria and the continued importance of vector control in managing infectious diseases [1,4]. Building on Ross's legacy, researchers continue to explore new methods to combat malaria, such as genetic modification of mosquitoes to reduce transmission, development of malaria vaccines, and novel antimalarial drugs influence on global health policies. Ross's contributions extended beyond malaria control to influence broader public health policies [14]. His work highlighted the importance of sanitation, preventive measures, and public health education in controlling infectious diseases. Ross's emphasis on evidence-based policy development and his role as a consultant to various governments and organizations helped shape global health policies aimed at improving disease prevention and control. His legacy is reflected in the ongoing efforts to address infectious diseases through comprehensive public health strategies. Ross was a strong advocate for integrated public health approaches that combined scientific research, policy development, and community engagement. He recognized that effective disease control required a multidisciplinary effort involving scientists, policymakers, and the public. Ross's advocacy for such holistic approaches has influenced contemporary public health strategies, which emphasize the importance of collaboration and community involvement in addressing health challenges [12]. Ross's development of mathematical models to describe the dynamics of malaria transmission was a pioneering achievement in the field of epidemiology. His models provided a theoretical framework for understanding how diseases spread within populations and how various factors, such as vector density and human behavior, influence transmission. These early models laid the groundwork for modern epidemiological modeling, which continues to be a critical tool in predicting and controlling disease outbreaks. Ross's integration of empirical research with mathematical modeling remains a cornerstone of epidemiological studies. Ross's contributions to medical education are significant. He authored several influential books and papers that disseminated his research findings and methodologies to a broader audience. His works, such as "The Prevention of Malaria" (1910), provided comprehensive guides to understanding and controlling malaria, serving as valuable resources for medical professionals and researchers [18]. Ross's emphasis on rigorous scientific training and his role in shaping curricula for tropical medicine courses have had a lasting impact on medical education. Throughout his career, Ronald Ross has received numerous accolades and honors in recognition of his pioneering work. In 1902, he was awarded the Nobel Prize in Physiology or Medicine, becoming the first British Nobel laureate. He was knighted in 1911 and elected as a Fellow of the Royal Society in 1901. These honors reflect the significant contributions Ross made to science and public health. Memorials and plaques commemorating Ross's achievements can be found in various locations, including Secunderabad, India, where he made his groundbreaking discovery, and London, where his contributions to medical science are celebrated. The impact of Ross's discoveries extended beyond immediate malaria control measures. His work influenced global health policies and the development of tropical medicine as a distinct field [19].

Founding of tropical medicine institutions: Ross's achievements contributed to the establishment of institutions dedicated to tropical medicine, such as the Liverpool School of Tropical Medicine, where he served as the first professor of tropical medicine. These institutions played a crucial role in advancing research and training healthcare professionals in tropical diseases.

Shaping international health organizations: Ross's work helped shape the policies and strategies of international health organizations, including the World Health Organization (WHO). His emphasis on vector control and public health interventions became integral components of global malaria eradication efforts [20].

Advancing research and development: Ross's findings stimulated further research into the biology and epidemiology of malaria, leading to the development of new diagnostic tools, treatments, and preventive measures. His work paved the way for modern malaria research and control programs.

Ross's findings had a profound impact on public health, leading to strategies focused on mosquito control to combat malaria. His work laid the groundwork for future research and prevention measures, including the use of insecticides and bed nets, which have saved countless lives. After his landmark discoveries, Ross continued his work in tropical medicine, holding prominent academic positions, and contributing to the mathematical modeling of malaria transmission. His interdisciplinary approach and dedication to public health have left a lasting legacy in the fields of parasitology and tropical medicine. Ronald Ross's pioneering research not only revolutionized the understanding of malaria but also established a foundation for ongoing global efforts to eradicate the disease. His achievements underscore the importance of scientific inquiry and its potential to transform public health.

Discussion

Sir Ronald Ross's work represents a significant paradigm shift in the understanding and control of malaria, a disease that has plagued humanity for millennia [1]. Before Ross's groundbreaking discoveries, the prevailing theory of disease transmission was the miasma theory, which posited that diseases such as malaria were spread by "bad air" emanating from decaying organic matter. Ross's meticulous research and subsequent discovery of the malaria parasite's lifecycle within the mosquito fundamentally changed this perspective and paved the way for modern vector control strategies. Ross's work provided concrete evidence that malaria was not caused by miasma but by a protozoan parasite transmitted by the Anopheles mosquito [21]. This discovery was crucial in shifting the focus of malaria research and control from generalized sanitary measures to targeted interventions aimed at the mosquito vector. By identifying the mosquito as the critical link in malaria transmission, Ross's findings highlighted the importance of understanding vector biology and behavior, which became essential for developing effective control strategies. Ross's methodologies were pioneering in their integration of field and laboratory research [22]. His approach combined detailed observational studies of mosquito behavior with rigorous laboratory experiments, where he dissected mosquitoes to trace the lifecycle of the malaria parasite. This dual approach not only provided empirical evidence for his theories but also set a methodological standard for future research in parasitology and tropical medicine. His collaborative efforts were also significant. Ross worked closely with local Indian medical staff and other researchers, demonstrating the value of cross-disciplinary and cross-cultural collaborations in scientific research. These partnerships were instrumental in conducting large-scale experiments and validating his findings across different geographic regions, ultimately leading to widespread acceptance of his theories. Initially, Ross's findings faced considerable skepticism from the scientific community. The miasma theory had been deeply entrenched for centuries, and the notion that tiny, invisible parasites could be responsible for such a widespread and deadly disease was difficult for many to accept. Despite these challenges, Ross remained steadfast in his research, meticulously gathering data and presenting his findings with clarity and conviction. His perseverance paid off when, in 1902, he was awarded the Nobel Prize in Physiology or Medicine [23]. This recognition not only validated his work but also helped to shift the scientific consensus toward the acceptance of vector-borne disease theories. The Nobel Prize served as a catalyst for further research into mosquito-borne diseases, encouraging other scientists to explore and expand upon Ross's groundbreaking work. The implications of Ross's discoveries were profound and far-reaching. By proving that mosquitoes were the vectors for malaria, Ross laid the groundwork for vector control as a primary strategy in malaria prevention. This led to the development and implementation of various public health measures, such as the use of insecticides, mosquito nets, and environmental management to reduce mosquito breeding sites. Ross's work also influenced the development of global health policies and malaria eradication programs. His emphasis on vector control became a cornerstone of malaria control strategies worldwide, guiding efforts to reduce the incidence of malaria in endemic regions [24]. These strategies have evolved over time but remain rooted in the fundamental principles established by Ross's research. The legacy of Ronald Ross extends beyond his immediate discoveries [25]. His work exemplifies the importance of scientific rigor, perseverance, and the willingness to challenge established theories. Ross's methodologies and collaborative approaches continue to serve as a model for contemporary research in parasitology and tropical medicine. In the context of modern public health, Ross's contributions remain highly relevant. Malaria continues to be a major global health challenge, particularly in sub-Saharan Africa and Southeast Asia [26]. The principles of vector control that Ross championed are still integral to current malaria control and eradication efforts. Innovations in insecticide-treated nets, indoor residual spraying, and environmental management all trace their conceptual origins to Ross's seminal work. Moreover, Ross's emphasis on understanding the lifecycle and behavior of disease vectors has inspired similar approaches in controlling other vector-borne diseases, such as dengue fever, Zika virus, and West Nile virus [27]. His work underscores the enduring importance of integrating field and laboratory research, fostering collaboration, and maintaining a steadfast commitment to scientific inquiry in the face of skepticism and adversity [28]. Ronald Ross's contributions to malaria research have had a transformative impact on public health [29]. His discovery of the mosquito transmission of malaria revolutionized disease control strategies, set new standards for scientific research, and continues to influence contemporary efforts to combat vector-borne diseases.

## Conclusions

Ronald Ross's pioneering work in identifying the mosquito as the vector for malaria revolutionized the understanding and control of one of the world's most devastating diseases. His legacy endures in the sustained efforts to combat malaria and in the broader field of parasitology and tropical medicine. Ross's achievements serve as a testament to the impact of dedicated scientific inquiry and the enduring quest to improve public health. The legacy of Ronald Ross is multifaceted. He exemplified the role of a scientist dedicated to solving real-world problems, emphasizing the need for persistent inquiry and collaboration across disciplines. His work continues to inspire current and future generations of scientists, researchers, and public health professionals. The ongoing fight against malaria and other vector-borne diseases still relies on the principles he established over a century ago. As we continue to build on his legacy, Ross’s work serves as a reminder of the profound impact that dedicated scientific endeavors can have on human health and well-being.
